# Development of bullous systemic lupus erythematosus in patient treated with NB‐UVB: A case report and comprehensive review of the literature

**DOI:** 10.1002/ccr3.9037

**Published:** 2024-05-31

**Authors:** Elham Behrangi, Alireza Jafarzadeh, Abbas Dehghani, Nasrin Shayanfar, Azadeh Goodarzi

**Affiliations:** ^1^ Department of Dermatology, Rasool Akram Medical Complex Clinical Research Development Center (RCRDC), School of Medicine Iran University of Medical Sciences (IUMS) Tehran Iran; ^2^ Skin and Stem Cell Research Center Tehran University of Medical Sciences Tehran Iran; ^3^ Department of Pathology, Rasool Akram Medical Complex, School of Medicine Iran University of Medical Sciences Tehran Iran

**Keywords:** bullous systemic lupus erythematosus, case report, phototherapy, systemic lupus erythematosus, UVB, vesiculobullous lesion

## Abstract

**Key Clinical Message:**

The use of phototherapy is highly effective in treating various skin diseases. In this study, the aim is to present vesicular and blister lesions in patients treated with UVB for psoriasis. It is advisable to consider the possibility of BSLE in cases of vesiculobullous lesions following phototherapy, along with other potential diagnoses.

**Abstract:**

Bullous systemic lupus erythematosus (BSLE) is a rare form of cutaneous lupus erythematosus that presents as vesicles and blisters on various parts of the body. The pathological appearance of these lesions often shows subepidermal vesicles with deposits of IgG, IgM, IgA, and complement C3 in granular or linear forms under direct immunofluorescence (DIF) examination. Clinical studies demonstrate the effectiveness of phototherapy in treating various skin conditions. While several studies suggest a correlation between phototherapy and the development of vesiculobullous lesions, most of these reports are related to bullous pemphigoid, with limited research on the occurrence of BSLE following phototherapy. In this case report, vesicular and blistering lesions in a 70‐year‐old man undergoing UVB treatment for psoriasis are described. Pathological examination confirmed the diagnosis of bullous systemic lupus erythematosus, and the patient experienced significant improvement after treatment with dapsone tablets. A literature review was conducted on the development of vesiculobullous lesions after phototherapy, comparing different approaches presented in previous studies. Our conclusion highlights the importance of considering BSLE as a possible diagnosis in cases of vesiculobullous lesions post‐phototherapy, alongside other potential conditions.

## 1 INTRODUCTION

Systemic lupus erythematosus (SLE) is a chronic autoimmune disease characterized by the production of autoantibodies against nuclear and cytoplasmic antigens, which affect several organs such as the kidneys, skin, and joints.^1^ Bullous systemic lupus erythematosus (BSLE) is known as a rare skin manifestation of systemic lupus erythematosus (SLE) and is present in less than 1% of patients with SLE. This condition presents with numerous tense blisters and vesicles on various parts of the body.^2^, ^3^ While all body regions can be affected, the upper trunk, axilla, proximal limbs, and neck are commonly involved. Other clinical manifestations in patients may include the development of urticarial papules, erythematous plaques, and targetoid lesions. Itching is typically not severe in these patients, and they often describe a burning sensation.^4^ Histopathologically, the identification of subepidermal blistering accompanied by lymphocyte and neutrophil infiltration in the periadnexal and perivascular regions is a diagnostic hallmark of the disease. Additionally, the presence of IgG, IgA, and IgM deposition, along with complement, in the epidermal basement membrane on direct immunofluorescence examination contributes to the diagnostic process.^5^, ^6^, ^7^

Phototherapy stands out as a highly effective treatment modality for various dermatological conditions such as psoriasis, vitiligo, morphea, parapsoriasis, and mycosis fungoides.^8^, ^9^, ^10^, ^11^, ^12^, ^13^ Ultraviolet (UV) light, possessing shorter wavelengths and higher energy levels than visible light, can penetrate the epidermis and dermis to exert therapeutic effects. UV light is categorized into three types based on wavelength: UVA, UVB, and UVC.^14^, ^15^, ^16^

## 2 CASE HISTORY/EXAMINATION

**FIGURE 1:**
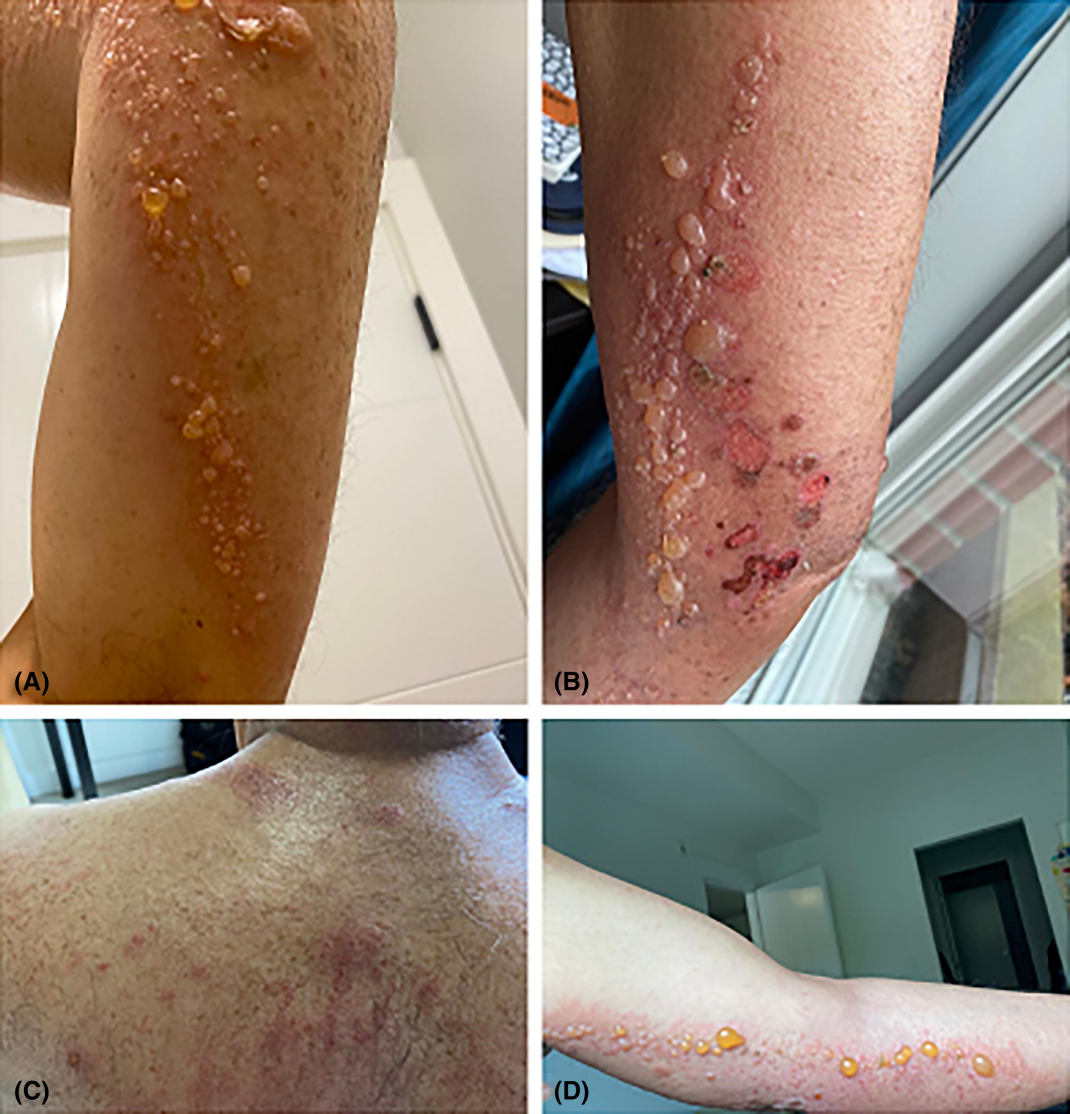
(A–D), Clinical features of lesions. (A, B) Vesicles and bullae on the lower limbs. (C) erythematous papules on the back. (D) Vesicles and bullae on the upper limb.

A 70‐year‐old Iranian man visited our clinic presenting with multiple pruritic blisters that had appeared all over his body 6 months prior. His medical history encompassed psoriatic lesions over a span of 25 years, during which he had been using prednisolone tablets, clobetasol ointment, and a combination gel of calcipotriol/betamethasone. Upon physical examination, erythematous and scaly plaques were observed on the trunk, lower limbs, and elbows, alongside numerous blisters on the upper and lower limbs and trunk (Figure 1). Additionally, the patient's history includes photosensitivity in the facial area noted along with exposure to sunlight, joint pain in the knees, wrists, and ankles, as well as hair loss that was not related to scarring upon examination.

Eight months ago, UVB treatment was initiated twice a week due to the recurrence of psoriatic lesions. Following the protocol, the duration of irradiation was gradually increased at each session. After the 15th irradiation session, multiple papules and vesicles emerged across the body, progressively growing in number and size. The patient received treatment with 50 mg of prednisolone (with a weekly dose reduction) and clobetasol ointment. These interventions partially improved the lesions initially, but a recurrence occurred after 2 months, prompting a reiteration of the previous treatment. Despite partial improvement, the lesions resurfaced after 2 months, leading the patient to seek care at our clinic.

## 3 METHODS

**FIGURE 2:**
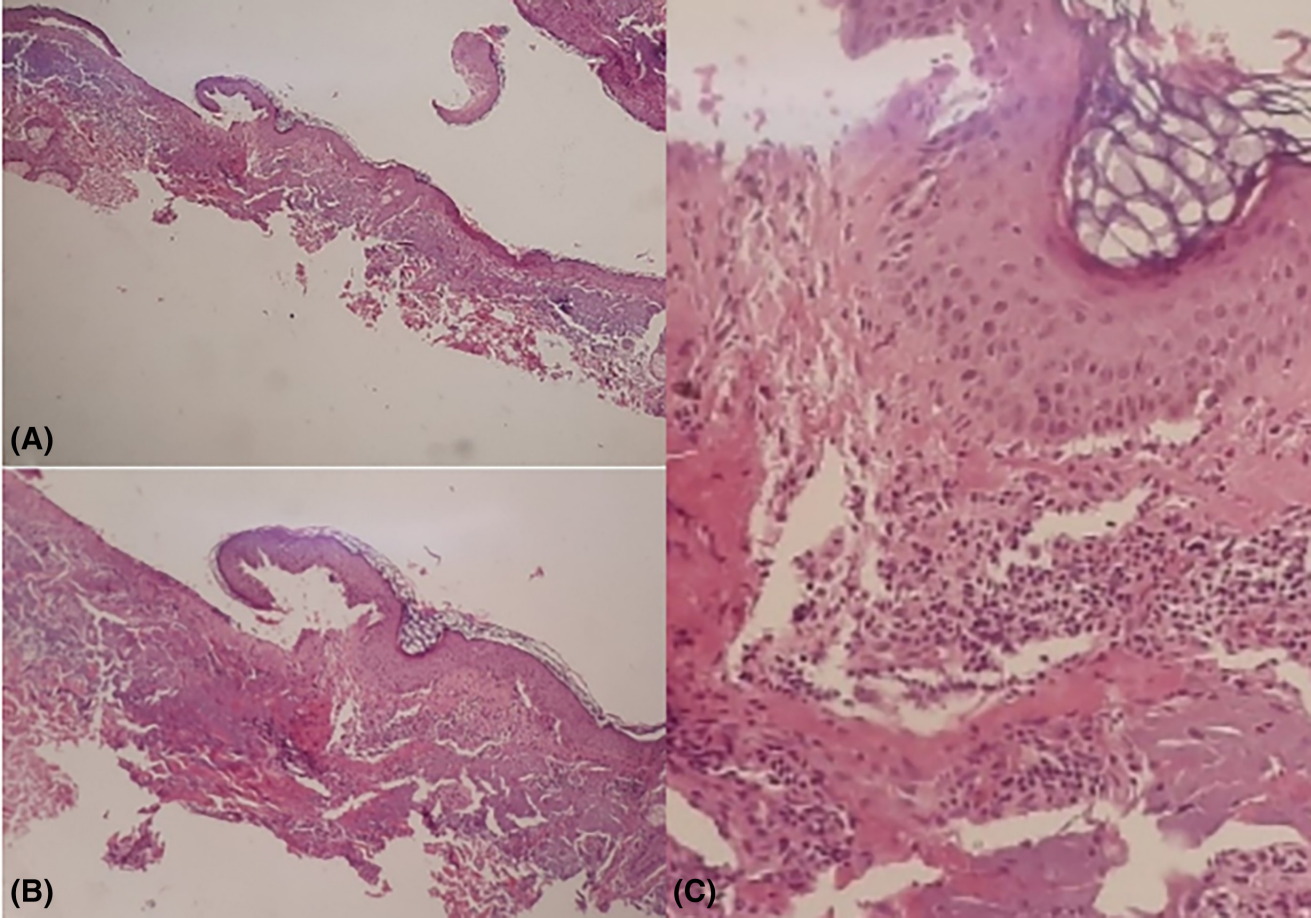
(A–C), Pathological appearance of lesion with Hematoxylin and eosin staining. subepidermal, neutrophil‐rich blister with superficial and mid dermal perivascular and perifollicular inflammation.

Following a thorough history‐taking and physical examination, the patient underwent a biopsy of the lesion and a direct immunofluorescence examination due to differential diagnoses of bullous pemphigoid and bullous lupus erythematosus. The dermatopathologist's report indicated a subepidermal blister rich in neutrophils, along with superficial and mid‐dermal perivascular and perifollicular inflammation (Figure 2). The direct immunofluorescence study confirmed linear deposition of IgG along the basement membrane zone, supporting the diagnosis of “bullous systemic lupus erythematosus.”

Laboratory tests conducted on the patient, including liver function tests, lipid profile tests, complete blood count, and serum glucose‐6‐phosphate dehydrogenase level, all returned normal results. Additionally, the serum levels of complement (C3, C4), CH50, anti‐ds DNA, ESR, and CRP were within normal ranges. The antinuclear antibody (ANA) level was reported as 1/160 (reference range: up to 1.80), and the rheumatoid factor (RF) level was 16 (reference range: up to 14). Furthermore, to rule out the diagnosis of bullous pemphigoid, indirect immunofluorescence and ELISA tests for Anti‐BP180 and anti‐BP230 were conducted, yielding negative results. The patient was treated with dapsone tablets at a daily dose of 50 mg.

## 4 CONCLUSION AND RESULTS

Following the administration of the medication, the skin lesions and itching showed significant improvement, and no new lesions were observed during the 1‐month and 2‐month follow‐ups. Furthermore, the patient reported no specific complaints.

## 5 DISCUSSION

To review similar studies, three databases—Scopus, PubMed, and Web of Science—were utilized. The keywords searched were “lupus”, “bullous systemic lupus erythematosus”, “SLE”, “BSLE”, “vesiculobullous lesions”, “bullous pemphigoid”, “psoriasis”, along with the terms “phototherapy”, “UVB”, “NB‐UVB”, “UVA”, and “BB‐UVB”. The inclusion criteria encompassed all types of clinical trials, case reports, and case series, while review studies, nonhuman studies, articles in languages other than English, and articles lacking full‐text were excluded. Ultimately, data from six studies were extracted for further analysis.

Bullous systemic lupus erythematosus (BSLE) is recognized as a rare skin disease. The clinical manifestations of the disease range from small vesicles to very large blisters.^3^, ^5^, ^17^ Histopathological observations typically demonstrate the presence of subepidermal blisters with a dense accumulation of neutrophils in dermal papillae, along with the infiltration of neutrophils and lymphocytes in perivascular and periadnexal areas. Linear, granular, or mixed deposits of IgG, IgA, IgM, and complement are also diagnostic findings upon examination of direct immunofluorescence (DIF).^4^, ^5^, ^18^

In this context, the diagnostic criteria outlined by Camisa and Sharma for diagnosing BSLE encompass: meeting the SLE diagnosis criteria set by the American College of Rheumatology; the presence of vesicles and bullae not confined to sun‐exposed skin; histopathology consistent with dermatitis herpetiformis; absence of circulating basement membrane zone (BMZ) antibodies in indirect immunofluorescence; and detection of IgG, and/or IgM, and frequently IgA at the BMZ in direct immunofluorescence.^19^, ^20^ Based on the study by Gammon and Briggaman, there appear to be at least two immunologically distinct subtypes of bullous SLE characterized by the presence or absence of circulating and/or tissue‐bound basement membrane zone autoantibodies that recognize type VII collagen.^21^

The formation of vesiculobullous lesions after phototherapy has been documented in a few studies, with these lesions reported post both UVB and UVA radiation exposures (Table 1). Among the reported cases, most lesions were associated with bullous pemphigoid, and instances of bullous systemic lupus erythematosus and lichen planus pemphigoides in connection with phototherapy have also been reported.^3^, ^22^, ^23^, ^24^, ^25^

**TABLE 1:** Comparison of reported vesiculobullous lesions after phototherapy.

Variables	Our study	Nasongkhla study (3)	Elizabeth study (22)	Barnadas study (23)	Chan study (24)	George study (25)	Ceryn study (29)
Gender	Male	Female	Male	Female	Female	Male	Male
Age(years)	70	51	50	65	54	61	71
Underlying disease	Psoriasis	None	Psoriasis	Psoriasis	Psoriasis	Psoriasis	Psoriasis
Type of phototherapy	NB‐UVB	UVB	PUVA	PUVA	NB‐UVB	PUVA	NB‐UVB
The time interval between the receipt of phototherapy and the development of a lesion	After the 15th session of phototherapy	48 h after the photo test	After the 18th session of phototherapy	After the 30th session of phototherapy	The fourth week of phototherapy	After the 15th session of phototherapy	10 weeks after the start of phototherapy.
Diagnosis	Bullous systemic lupus erythematosus	Bullous systemic lupus erythematosus.	Bullous pemphigoid	Bullous pemphigoid	Lichen planus pemphigoides	Bullous pemphigoid	Bullous pemphigoid
Treatment	Dapsone 50 mg/day p.o.	Dapsone 50 mg/day p.o.	0.1% triamcinolone	Prednisone 60 mg/day p.o.	Prednisolone at 0.5 mg/kg/day p.o.	Fluocinonide	Methotrexate
		Prednisone 30 mg/day p.o.	acetonide cream.			ointment	5 mg/week, i.m.

In our study, the occurrence of bullous systemic lupus erythematosus was linked to the administration of NB‐UVB treatment in a patient with psoriasis. Similarly, the study by Nasongkhla et al. highlighted the development of bullous systemic lupus erythematosus in regions exposed to UV radiation. The investigation, focusing on a patient with a possible diagnosis of dermatitis herpetiformis, showed that UVB exposure led to blistering lesions. Biopsy results and direct immunofluorescence confirmed the diagnosis of bullous systemic lupus erythematosus. Notably, no blistering was observed in areas treated with UVA. The patient was managed with daily dapsone tablets at 50 mg, along with prednisolone at 30 mg, resulting in significant improvement of the lesions.^3^

Previous studies have discussed the role of UV radiation, particularly UVB radiation, in the pathogenesis of systemic lupus erythematosus.^26^, ^27^

One of the mechanisms proposed regarding the impact of UVB radiation on cutaneous lupus erythematosus occurrence is the apoptosis of keratinocytes. This process leads to incomplete removal by macrophages, resulting in cell necrosis, release of pro‐inflammatory factors, exposure of cellular antigens to the immune system, and subsequent skin manifestations of the disease.^28^ Another suggested mechanism involves antibody‐dependent cellular cytotoxicity, where serum levels of lupus‐related antibodies rise, leading to the manifestation of skin disease symptoms.^26^, ^27^

However, studies and reports on the occurrence of BSLE after phototherapy are very limited. Further studies are needed to confirm the results observed in our study. Moreover, in our study and in the study by Nasongkhla et al., the occurrence of BSLE happened following UVB exposure. Therefore, it is imperative to investigate if UVA radiation can also lead to similar lesions. If the lesions are solely related to UVB treatment, patients should not be excluded from UVA therapy.

Despite the scarcity of case reports on BSLE associated with UVB exposure, other vesiculobullous disorders have been reported in the context of phototherapy. A study by Chan et al. documented the occurrence of lichen planus pemphigoides in a patient undergoing NB‐UVB phototherapy for guttate psoriasis.^24^ In a 2022 study by Ceryn et al., bullous pemphigoid was reported in a psoriasis patient treated with NB‐UVB for 2 months.^29^ Additionally, George (1995),^25^ Barnadas (2006),^23^ and Elizabeth (1979)^22^ reported bullous pemphigoid in psoriasis patients treated with Psoralen plus UVA (PUVA).

The underlying condition in our patient and in most cases of vesiculobullous lesions post‐phototherapy was psoriasis. This case suggests that psoriasis is one of the most common indications for phototherapy. Conversely, dysregulation of T‐cell activity in psoriasis might trigger autoantibodies against the basement membrane.^30^

The treatment strategy for vesiculobullous lesions post‐phototherapy involves discontinuing the treatment and initiating specific therapies for each lesion, as observed in previous studies.^3^, ^23^, ^24^, ^25^, ^29^ The patient in our study received daily dapsone tablets at 50 mg while phototherapy was halted. Follow‐up examinations at 1 and 2 months revealed significant improvement.

Dapsone is not currently FDA‐approved for treating BSLE.^31^ Nevertheless, its efficacy has been demonstrated in numerous studies.^3^, ^31^, ^32^, ^33^, ^34^ Administering dapsone at a dosage of 2 mg/kg/day has shown significant improvement in BSLE lesions. Additionally, lower doses of dapsone (25–50 mg daily) have also been linked to treatment response and lesion improvement.^17^ The improvement in lesions with dapsone is likely attributed to the modulation of neutrophil and lymphocyte responses at the lesion site, as well as the reduction in their migration.^31^, ^32^

## 6 CONCLUSION

This study reported a case of bullous lupus following treatment with NB‐UVB, showing significant improvement with dapsone tablet therapy. Consequently, it is advisable to consider this diagnosis for blistering lesions post‐UVB treatment in patients. A limitation of our study is its small sample size and reporting on a single patient; hence, it is recommended that future studies undertake a more comprehensive investigation with a larger sample size.

### 6.1 Recommendation

It is recommended that the appearance of vesicular lesions following phototherapy be considered a serious concern. Among other potential diagnoses, lupus bolus should be carefully evaluated. Following biopsy and diagnosis, the patient should receive appropriate treatment.

## AUTHOR CONTRIBUTIONS

**Elham Behrangi:** Conceptualization; writing – original draft; writing – review and editing. **Alireza Jafarzadeh:** Investigation; resources; validation; writing – review and editing. **Abbas Dehghani:** Investigation; writing – original draft. **Nasrin Shayanfar:** Data curation; investigation; methodology; validation; writing – review and editing. **Azadeh Goodarzi:** Investigation; project administration; writing – original draft.

## FUNDING INFORMATION

None.

## ETHICS STATEMENT

Due to the research protocol at the Iran University of medical sciences, the ethical committee's approval for case reports is not needed; however, the patient's consent for publication was obtained.

## CONSENT

Written informed consent was obtained from the patient to publish this report in accordance with the journal's patient consent policy.

## TRANSPARENCY DECLARATION

Authors declare that the manuscript is an honest, accurate, and transparent. No important aspect of the study is omitted.

## Data Availability

All data produced in the present study are available upon reasonable request to the authors.
